# Correlates of poor glycemic control among patients with diabetes initiating hemodialysis for end-stage renal disease

**DOI:** 10.1186/s12882-015-0204-4

**Published:** 2015-12-09

**Authors:** Jinnie J. Rhee, Victoria Y. Ding, David H. Rehkopf, Cristina M. Arce, Wolfgang C. Winkelmayer

**Affiliations:** Division of Nephrology, Stanford University School of Medicine, 1070 Arastradero Road #3C3109, Palo Alto, CA 94304 USA; Division of Bioinformatics Research, Stanford University School of Medicine, Palo Alto, CA USA; Division of General Medical Disciplines, Department of Medicine, Stanford University School of Medicine, Palo Alto, CA USA; Division of Nephrology, University of Texas Southwestern Medical Center, Dallas, TX USA; Selzman Institute for Kidney Health, Section of Nephrology, Department of Medicine, Baylor College of Medicine, Houston, TX USA

**Keywords:** Diabetes mellitus, Electronic health records, End-stage renal disease, Glycemic control, Hemodialysis, USRDS

## Abstract

**Background:**

Maintaining tight glycemic control is important for prevention of diabetes-related outcomes in end-stage renal disease patients with diabetes, especially in light of their poor prognosis. This study aimed to determine factors associated with poor glycemic control among U.S. patients with diabetes mellitus initiating hemodialysis for end-stage renal disease.

**Methods:**

Using data from the U.S. Renal Data System, electronic health records of a large national dialysis provider, and U.S. Census data, we performed a cross-sectional multivariable Poisson regression analysis to characterize risk factors associated with poor glycemic control, defined as glycated hemoglobin (HbA1c) >7 vs. ≤7 %, in adult patients with diabetes who initiated hemodialysis at an outpatient facility between 2006 and 2011.

**Results:**

Of 16,297 patients with diabetes, 21.2 % had HbA1c >7 %. In multivariable analysis, younger patients, patients of Native American race, and those of Hispanic ethnicity had higher prevalence of poor glycemic control. Independent correlates of poor glycemic control further included higher platelet count, white blood cell count, and ferritin; higher body mass index, systolic blood pressure, total cholesterol and triglyceride concentrations; lower HDL and albumin concentrations; lower normalized protein catabolic rate; and higher estimated glomerular filtration rate at initiation of dialysis (all *P* < 0.05). No independent associations were found with area-level socioeconomic indicators. Occurrence of diabetes in patients <40 years of age, a proxy for type 1 diabetes, was associated with poor HbA1c control compared with that in patients ≥40 years of age, which was classified as type 2 diabetes. These findings were robust to the different outcome definitions of HbA1c >7.5 % and >8 %.

**Conclusion:**

In this cohort of incident end-stage renal disease patients with diabetes, poor glycemic control was independently associated with younger age, Native American race, Hispanic ethnicity, higher body mass index, and clinical risk factors including atherogenic lipoprotein profile, hypertension, inflammation, and markers indicative of malnutrition and a more serious systemic disease.

## Background

Diabetes mellitus is the leading cause of end-stage renal disease (ESRD) and accounts for 30–50 % of incident ESRD cases [1]. Previous studies have shown that patients with diabetes and ESRD requiring dialysis have higher rates of several comorbidities and experience poorer clinical outcomes compared with patients without diabetes. Glycemic control in these patients has been shown to be associated with microvascular and macrovascular complications and mortality [2–4]. Point-of-care glycated hemoglobin (HbA1c) is commonly used as a marker for long-term glycemic control that reflects average blood glucose concentration over approximately 3 months in patients with diabetes [5, 6]. Monitoring and improving glycemic control is essential to diabetes care and management in order to delay the progression of micro- and macrovascular complications related to diabetes [7]. Improved glycemic control may reduce the risk of myocardial infarction and cardiovascular death [8], and improve survival in dialysis patients with diabetes [9]. According to the Kidney Disease Outcomes Quality Initiative Clinical Practice Guideline for Diabetes and Chronic Kidney Disease, HbA1c levels of 7–9 % have been shown to be associated with better clinical outcomes in hemodialysis patients, though this relationship has not been supported by all observational studies, and data from prospective, randomized studies are lacking [10]. Still these guidelines suggest that patients with diabetes who are on dialysis may benefit from intensive glycemic control (HbA1c ≤ 7 %) due to reduction in the incidence of microvascular complications [10].

There are limited data on risk factors for suboptimal glycemic control in patients with diabetes requiring maintenance hemodialysis. Maintaining optimal glycemic levels is difficult in these patients because they often present with other comorbidities and clinical conditions that could contribute to poor glycemic control [11]. From a clinical and a health policy perspective, understanding the various sociodemographic and clinical factors impacting glycemic control in hemodialysis patients with diabetes is important, as this knowledge could help guide future studies and interventions to improve patient outcomes and access to quality care. In the present study, we aimed to determine factors associated with poor glycemic control in U.S. patients with diabetes whose declining kidney function mandated initiation of renal replacement therapy using hemodialysis.

## Methods

### Data source

We used data from the United States Renal Data System (USRDS), the national registry for patients with ESRD [12], and data from the electronic health records (EHR) of DaVita, Inc., the second largest national provider of dialysis services in the U.S. Information covering years 2006 to 2011 from both sources were merged using a crosswalk of anonymized patient identifiers generated by the USRDS Coordinating Center, with approval by the Centers for Medicare and Medicaid Services (CMS) and the National Institutes of Diabetes and Digestive and Kidney Disease (NIDDK). The USRDS contains demographic data for almost all Americans with ESRD, data from final-action Medicare claims (Parts A, B, D) for eligible patients, as well as information on comorbidities. The DaVita EHR provides highly granular and longitudinal data on laboratory values including HbA1c, all measured centrally, as well as on vital signs and hemodialysis-related parameters, all measured at the point of care.

### Study population

Our study population included all adult patients (≥18 years old) with incident ESRD between 2006 and 2011 whose Medical Evidence Report (form CMS-2728) identified diabetes as a reported comorbidity or cause of kidney disease. Using information from the USRDS Condensed Treatment History (RXHIST60) file, we restricted the cohort to those who received their maintenance hemodialysis treatments at a DaVita outpatient facility and had no dialysis modality switches by day 90. We further restricted the cohort to those who had Medicare fee-for-service (Parts A + B) as their primary payer by 90 days after initiation of hemodialysis, and excluded patients with missing data on HbA1c, age, sex, race and ethnicity, and other covariates in the multivariable model. This study was approved by an institutional review board of Stanford University and conducted in accordance with the Declaration of Helsinki guidelines. Due to the unidentified nature of the data, patient consent was not deemed necessary.

### HbA1c measurements

We abstracted baseline HbA1c data obtained at the time the patient initiated hemodialysis at a DaVita outpatient facility (i.e., within 90 days of hemodialysis initiation) from the DaVita EHR. Poor glycemic control was defined as an HbA1c >7 %.

### Predictor variables

Information on age, sex, reported race (white, black, Asian, Native American, Pacific Islander, and other) and Hispanic ethnicity, and reported comorbidities were obtained from the Medical Evidence Report (form CMS-2728) in the USRDS. Oral antidiabetic treatment was ascertained from form CMS-2728 on which providers indicated whether a patient required insulin, oral antidiabetic drugs, or neither. Laboratory values, vital signs, and derived biometric parameters (normalized protein catabolic rate (nPCR); body mass index (BMI)) were abstracted from the EHR. No individual level socioeconomic data were available in USRDS or the EHRs. In order to address this lack of measures, which may be important for predicting poor glycemic control at baseline, we obtained area-level socioeconomic data from the U.S. Census Bureau American Community Survey (ACS). We were constrained to use ZIP code as the area of analysis in order to match the smallest indicator of geography available in the registry data. While for some outcomes ZIP code can lead to unstable associations due to heterogeneity of characteristics within ZIP code, for most outcomes examined, results have been shown to be consistent with smaller census-defined levels of geography [13]. ACS data were obtained for the following ZIP code characteristics (table number): median rent (B25064), median household income (B19013), percent below poverty (B06012), employment (B23001) and level of educational attainment (B15002) using the R package “acs” version 1.2 within the R computing environment.

### Statistical analysis

We described baseline patient characteristics using means and standard deviations for normally distributed continuous variables, medians and interquartile ranges for non-normally distributed data, and counts and proportions for categorical data for the overall cohort as well as for categories of low (<5.5 %), moderate (5.5 to <7 %), and high (≥7 %) levels of HbA1c.

We calculated prevalence ratios (PR) and their corresponding 95 % confidence intervals (CI) using multivariable Poisson regression with robust variance to characterize factors associated with poor glycemic control, which was defined as HbA1c >7 vs. ≤7 %. Variables were chosen a priori as potential determinants of poor glycemic control regardless of their statistical significance. Each patient’s estimated glomerular filtration rate (eGFR) was calculated from creatinine concentrations reported at initiation of dialysis using the 4-variable Modification of Diet in Renal Disease equation [14]. Age, platelet count, white blood cell count, and the five socioeconomic variables were assessed in tertiles. All other variables were modeled according to clinically relevant categories. We performed univariate analyses then two multivariable analyses: model 1 included demographic and area-level socioeconomic variables, and model 2 included all demographic, area-level socioeconomic, clinical and biometric variables. We conducted sensitivity analyses by redefining poorly controlled glycemic control as HbA1c >7.5 % and >8 %. We also performed stratified analyses by diabetes type. Data on specific type of diabetes are not available in the USRDS. As such, we used age as a surrogate for diabetes type and classified occurrence of diabetes in patients younger than age 40 as type 1 diabetes, and that in patients ≥40 years of age as type 2 diabetes. This approach has been used in previous studies [15]. Usually, we would conduct a series of tests for effect modification to formally identify any differences among individual associations between diabetes types. However, the proportion of patients with type 1 diabetes was found to be extremely small (4 %) and so any significant test for effect modification would have more likely represented false positive or chance findings rather than true differences in associations. Hence, we presented models stratified by diabetes type without formally motivating this action with tests for effect modification. All statistical tests were two-sided and conducted at the 0.05 level of significance, and all analyses were performed using SAS software package, version 9.4 (SAS Institute Inc., Cary, NC).

## Results

**Fig. 1:**
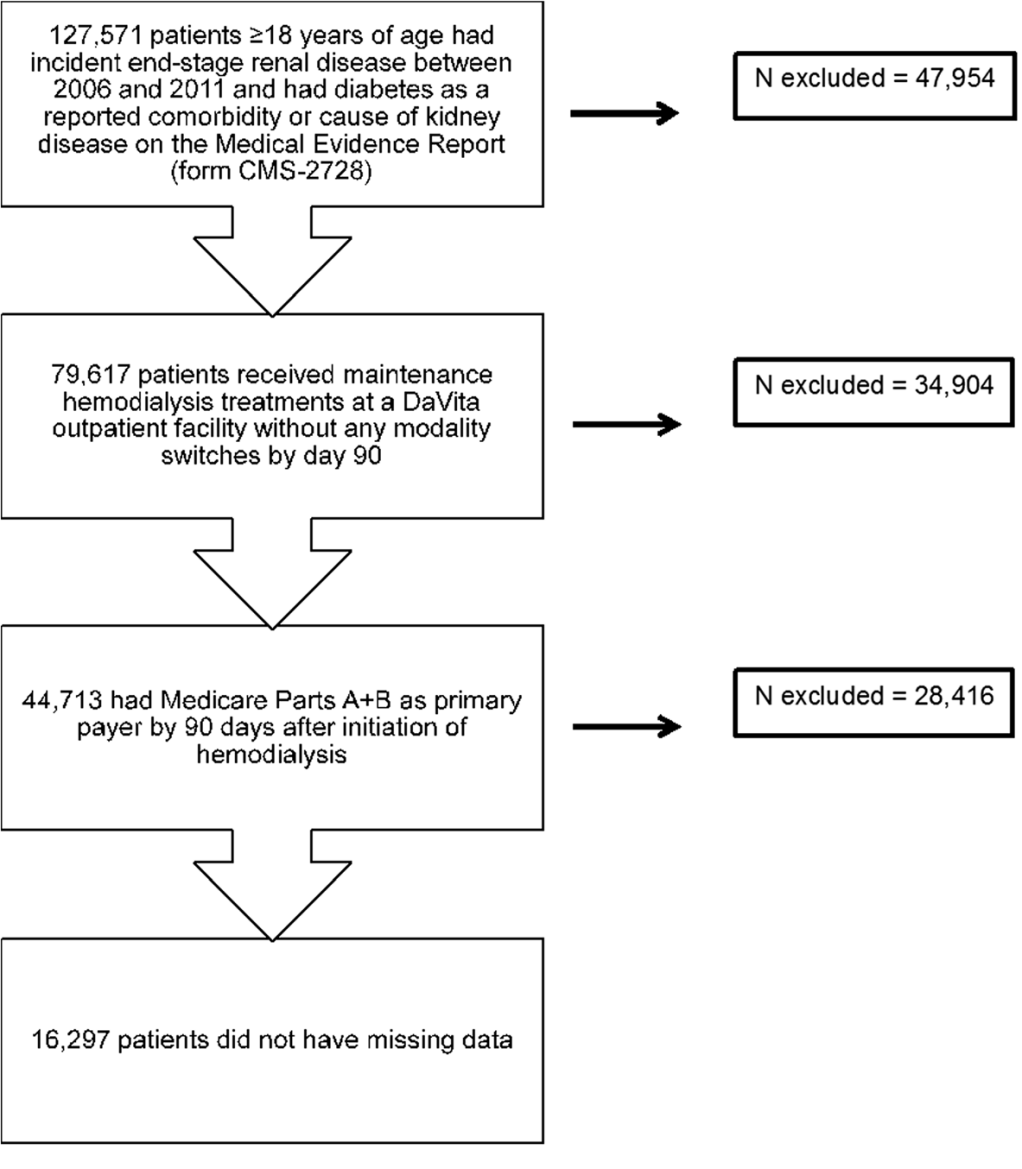
Study population from the US Renal Data System and electronic health records of DaVita, Inc. We selected a cohort of patients 18 years of age or older with incident ESRD between 2006 and 2011 and whose Medical Evidence Report (form CMS-2728) identified diabetes as a reported comorbidity or cause of kidney disease. We restricted the cohort to those who received their maintenance hemodialysis treatments at a DaVita outpatient facility and had no dialysis modality switches by day 90. We further restricted the cohort to those who had Medicare fee-for-service (Parts A + B) as their primary payer by 90 days after initiation of hemodialysis, and excluded patients with missing data on HbA1c, age, sex, race and ethnicity, and other covariates in the multivariable model

In the merged USRDS-DaVita crosswalk dataset, we identified 127,571 adult patients with incident ESRD between 2006 and 2011 with diabetes as a reported comorbidity or cause of kidney disease. Of those, 79,617 patients received maintenance hemodialysis treatments at a DaVita outpatient facility without a modality switch by day 90. After excluding those who did not have Medicare fee-for-service (Parts A + B) as their primary payer by 90 days after initiation of hemodialysis, and those with missing data on HbA1c, age, race and ethnicity, and other covariates, our final cohort included 16,297 patients (Fig. 1). The mean HbA1c in this cohort was 6.4 % and 21.2 % of patients had an HbA1c >7 %, with the mean HbA1c in this group being 8.2 %, indicating poorly controlled diabetes. Sociodemographic and clinical characteristics of the overall cohort as well as by HbA1c strata are shown in Table 1.

**Table 1:** Sociodemographic and clinical characteristics of US adult patients with diabetes mellitus initiating maintenance hemodialysis^a^

Variables		HbA1c in Categories			
	All patients	<5.5 %	5.5 to <7 %	≥7 %	*P*-value
	(*N* = 16,297)	(*N* = 3399)	(*N* = 9073)	(*N* = 3825)	
Patient characteristics					
HbA1c (%), mean ± SD	6.4 ± 1.6	5.0 ± 0.3	6.2 ± 0.4	8.0 ± 2.5	<0.001
Demographics					
Age (years), mean ± SD	64.7 ± 12.9	66.4 ± 12.6	65.8 ± 12.4	60.6 ± 13.4	<0.001
Male sex, *N* (%)	8817 (54.1)	1744 (51.3)	4997 (55.1)	2076 (54.3)	<0.001
Race, *N* (%)					<0.001
White	10,439 (64.1)	2158 (63.5)	5887 (64.9)	2394 (62.6)	
Native American	376 (2.4)	56 (1.7)	185 (2.0)	135 (3.5)	
Asian	483 (3.0)	108 (3.2)	274 (3.0)	101 (2.6)	
Black	4842 (29.7)	1049 (30.9)	2629 (29.0)	1164 (30.4)	
Pacific Islander	110 (0.7)	18 (0.5)	69 (0.8)	23 (0.6)	
Other/Multiracial	47 (0.3)	10 (0.3)	29 (0.3)	8 (0.2)	
Hispanic ethnicity, *N* (%)	2847 (17.5)	497 (14.6)	1552 (17.1)	798 (20.9)	<0.001
Reported comorbidities, *N* (%)					
Heart failure	6369 (39.1)	1251 (36.8)	3666 (40.4)	1452 (37.8)	<0.001
Atherosclerotic heart disease	3924 (24.1)	735 (21.6)	2316 (25.5)	873 (22.8)	<0.001
Cerebrovascular disease	1747 (10.7)	366 (10.8)	983 (10.8)	398 (10.4)	0.77
Peripheral vascular disease	2669 (16.4)	494 (14.5)	1531 (16.9)	644 (16.8)	0.005
Hypertension	14,486 (89.0)	3001 (88.3)	8082 (89.1)	3403 (89.0)	0.45
Amputation	743 (4.6)	137 (4.0)	417 (4.6)	189 (4.9)	0.17
Cancer	936 (5.7)	227 (6.7)	574 (6.3)	135 (3.5)	<0.001
Current tobacco use	899 (5.5)	186 (5.5)	473 (5.2)	240 (6.3)	0.05
Socioeconomic variables					
Median rent ($), mean ± SD	860.74 ± 272.49	867.67 ± 284.03	863.31 ± 271.87	848.47 ± 263.02	0.005
Median household income ($), mean ± SD	46,715 ± 16,970	46,569 ± 17,203	47,028 ± 17,107	46,102 ± 16,412	0.02
% below poverty, mean ± SD	17.9 ± 10.1	17.8 ± 10.0	17.6 ± 10.0	18.7 ± 10.3	<0.001
% unemployed, mean ± SD	6.3 ± 2.6	6.3 ± 2.7	6.2 ± 2.6	6.4 ± 2.5	0.007
% < high school education, mean ± SD	17.4 ± 10.8	16.9 ± 10.3	17.2 ± 10.7	18.4 ± 11.3	<0.001
Antidiabetic medication use, *N* (%)					
Insulin	10,676 (65.5)	1795 (52.8)	5886 (64.9)	2995 (78.3)	<0.001
Oral antidiabetic medications	4404 (27.0)	1141 (33.6)	2499 (27.5)	764 (20.0)	<0.001
Both insulin and oral antidiabetic medications	427 (4.0)	71 (4.0)	230 (3.9)	126 (4.2)	<0.001
No medications	1528 (9.4)	519 (15.3)	853 (9.4)	156 (4.1)	<0.001
Laboratory measurements					
Body mass index (kg/m^2^), mean ± SD	30.3 ± 7.8	29.6 ± 7.6	30.4 ± 7.8	30.7 ± 8.0	<0.001
Platelet count (×10^3^/μL), median (25th–75th percentile)	236.0 (186.0–296.0)	226.0 (174.0–285.0)	235.0 (186.0–294.0)	245.0 (198.0–311.0)	<0.001
White blood cell count (x1000/mm^3^), mean ± SD	7.6 ± 2.6	7.3 ± 2.6	7.6 ± 2.6	7.9 ± 2.7	<0.001
Ferritin (ng/mL), mean ± SD	365.5 ± 342.8	370.0 ± 347.1	373.4 ± 343.9	343.1 ± 335.4	<0.001
Total cholesterol (mg/dL), median (25th–75th percentile)	143.0 (118.0–173.0)	138.0 (115.0–166.0)	141.0 (116.0–171.0)	153.0 (126.0–185.0)	<0.001
LDL (mg/dL), median (25th–75th percentile)	72.0 (52.0–96.0)	70.0 (51.0–93.0)	71.0 (51.0–95.0)	77.0 (56.0–103.0)	<0.001
HDL (mg/dL), median (25th–75th percentile)	38.0 (31.0–48.0)	39.0 (31.0–49.0)	38.0 (30.0–47.0)	39.0 (32.0–50.0)	<0.001
Triglycerides (mg/dL), median (25th–75th percentile)	135.0 (96.0–191.0)	123.0 (89.0–172.0)	135.0 (96.0–190.0)	148.0 (104.0–214.0)	<0.001
Systolic blood pressure (mmHg), median (25th–75th percentile)	150.0 (132.0–169.0)	147.0 (129.0–167.0)	149.0 (131.0–168.0)	153.0 (135.0–173.0)	<0.001
Diastolic blood pressure (mm Hg), median (25th–75th percentile)	75.0 (65.0–87.0)	75.0 (64.0–85.0)	75.0 (64.0–86.0)	78.0 (67.0–90.0)	<0.001
Albumin (g/dL), mean ± SD	3.6 ± 0.5	3.5 ± 0.5	3.6 ± 0.5	3.5 ± 0.4	<0.001
Normalized protein catabolic rate (g/kg/d), mean ± SD	0.91 ± 0.32	0.88 ± 0.28	0.92 ± 0.32	0.93 ± 0.32	<0.001
Estimated GFR^b^ (mL/min/1.73 m^2^), mean ± SD	11.4 ± 4.8	10.9 ± 4.9	11.4 ± 4.7	11.7 ± 4.7	<0.001
Hemoglobin (g/dL), mean ± SD	12.1 ± 1.5	12.0 ± 1.6	12.1 ± 1.5	12.3 ± 1.5	<0.001

^a^Variables are described using means and standard deviations for normally distributed continuous data, medians and 25th and 75th percentile values for non-normally distributed data, and counts and proportions for categorical data

^b^GFR, glomerular filtration rate

The mean age of patients in this cohort was 64.7 years. Younger patients had higher HbA1c levels. The proportions of men, Native Americans, Hispanics, and current smokers increased in higher HbA1c categories. Several comorbidities, except for cerebrovascular disease, hypertension, and amputation, were more common in patients with HbA1c between 5.5 and <7 % compared with those with HbA1c <5.5 %. High HbA1c (>7 %) was correlated with lower prevalence of cancer. Between low (≤5.5 %) and moderate (5.5 to <7 %) HbA1c categories, there were only small quantitative differences in area-level median rent and prevalence of individuals living below poverty, unemployed, and those with less than high school education. However, in the HbA1c ≥7 % category, patients lived in areas with lower median rent, and slightly higher proportions lived under poverty and had less than high school education.

In this cohort, 65.5 % of patients were insulin users, 27.0 % were on oral antidiabetic medications; 4.0 % were on both insulin and oral antidiabetic medications, whereas 9.4 % of the patients were on neither treatment. The prevalence of insulin users as well those who used both insulin and other oral antidiabetic medications increased with higher levels of HbA1c, but the use of other oral antidiabetic medications alone was more common at low and moderate HbA1c levels.

Patients with higher HbA1c differed from those with better glycemic control in terms of all clinical parameters (all *P* < 0.05). Higher HbA1c was associated with higher BMI, higher platelet and white blood cell counts, higher lipid concentrations, blood pressure values, and higher nPCR and eGFR. Higher HbA1c was associated with lower ferritin concentrations.

The detailed results from the multivariable regression on the outcome of uncontrolled diabetes (HbA1c >7 %) are shown in Table 2. Compared with patients aged 57 years or younger, those aged 58–68 years had a 25 % (95 % CI, 19 %, 30 %) and those aged 69 years or older had a 47 % (95 % CI, 41 %, 52 %) lower prevalence of poor glycemic control. Native American patients had a 42 % (95 % CI, 17 %, 72 %) higher prevalence of poor control compared with non-Hispanic whites, whereas patients of Hispanic ethnicity had a 14 % (95 % CI, 3 %, 26 %) higher prevalence of poor glycemic control compared with non-Hispanic whites. No associations were identified between HbA1c control and sex, any of the other race categories, or current smoking status. Poor glycemic control was not associated with any of the five area-level socioeconomic factors, and adjusting for variables on the potential causal pathway between these socioeconomic factors and poor glycemic control did not substantially change the findings.

**Table 2:** Prevalence ratios of poor glycemic control (HbA1c >7 % vs. ≤7 %)^a^

Determinants	Unadjusted models	Model 1^b^	Model 2^c^	*P*-value^d^
Age (years)				
≤ 57	1	1	1	
58–68	0.69 (0.64, 0.74)	0.70 (0.65. 0.75)	0.75 (0.70, 0.81)	<0.001
≥ 69	0.47 (0.43, 0.51)	0.48 (0.44, 0.53)	0.53 (0.48, 0.59)	<0.001
Male sex	1.01 (0.94, 1.08)	0.96 (0.90, 1.03)	1.07 (0.99, 1.15)	0.06
Race/ethnicity^e^				
Native American	1.56 (1.30, 1.88)	1.41 (1.17, 1.71)	1.42 (1.17, 1.72)	<0.001
Asian	0.89 (0.72, 1.10)	0.94 (0.76, 1.17)	0.97 (0.78, 1.21)	0.78
Black	1.04 (0.97, 1.12)	1.01 (0.92, 1.10)	1.02 (0.93, 1.12)	0.64
Pacific Islander	0.96 (0.63, 1.47)	0.87 (0.57, 1.33)	0.93 (0.61, 1.43)	0.75
Other/Multiracial	0.82 (0.41, 1.64)	0.80 (0.40, 1.59)	0.75 (0.38, 1.50)	0.41
Hispanic ethnicity	1.29 (1.19, 1.40)	1.16 (1.05, 1.28)	1.14 (1.03, 1.26)	0.01
Median rent ($)				
≤ 735	1	1	1	
736–959	0.93 (0.86, 1.01)	0.92 (0.84, 1.01)	0.93 (0.85, 1.01)	0.10
≥ 960	0.94 (0.86, 1.02)	0.95 (0.86, 1.05)	0.98 (0.88, 1.08)	0.68
Median household income ($)				
< 38,629	1	1	1	
38,630–52,301	0.99 (0.91, 1.08)	1.12 (0.98, 1.28)	1.09 (0.98, 1.20)	0.11
> 52,303	0.93 (0.86, 1.01)	1.08 (0.98, 1.20)	1.11 (0.97, 1.27)	0.13
% below poverty				
< 12	1	1	1	
12–21	1.15 (1.05, 1.25)	1.07 (0.96, 1.19)	1.06 (0.95, 1.19)	0.28
≥ 21	1.22 (1.13, 1.33)	1.09 (0.94, 1.26)	1.09 (0.94, 1.27)	0.24
% unemployed				
< 5	1	1	1	
5–7	1.08 (0.99 1.18)	1.04 (0.96, 1.14)	1.04 (0.95, 1.13)	0.39
> 7	1.14 (1.05, 1.24)	1.07 (0.97, 1.18)	1.06 (0.97, 1.17)	0.21
% with < high school education				
< 11.5	1	1	1	
11.5–19.5	1.06 (0.97, 1.15)	0.94 (0.85, 1.04)	0.95 (0.86, 1.05)	0.29
≥ 19.5	1.20 (1.10, 1.30)	0.97 (0.86, 1.09)	0.97 (0.87, 1.10)	0.66
Smoking (current)	1.13 (0.98, 1.30)	---	0.98 (0.85, 1.13)	0.81
BMI (kg/m^2^)				
19- < 25	1	---	1	
25–29	0.99 (0.91, 1.10)	---	1.01 (0.92, 1.12)	0.78
≥ 30	1.16 (1.06, 1.27)	---	1.10 (1.01, 1.21)	0.04
Platelet count (×10^3^/μL)				
≤ 207	1	---	1	
208–277	1.23 (1.13, 1.34)	---	1.10 (1.01, 1.20)	0.04
≥ 278	1.41 (1.30, 1.53)	---	1.15 (1.05, 1.26)	0.003
White blood cell count (1000 per μL)				
< 6.3	1	---	1	
6.3–8.2	1.20 (1.10, 1.30)	---	1.11 (1.02, 1.21)	0.02
> 8.2	1.33 (1.22, 1.44)	---	1.18 (1.08, 1.29)	<0.001
Ferritin (ng/mL)				
< 200	1	---	1	
200–399	1.04 (0.96, 1.14)	---	1.02 (0.93, 1.11)	0.67
≥ 400	1.25 (1.16, 1.36)	---	1.17 (1.08, 1.27)	<0.001
Total cholesterol (mg/dL)				
≤ 200	1	---	1	
201–239	1.42 (1.28, 1.59)	---	1.12 (0.97, 1.26)	0.13
≥ 240	1.65 (1.44, 1.89)	---	1.12 (1.01, 1.29)	0.04
HDL (mg/dL)				
≤ 40	1	---	1	
40–59	0.79 (0.71, 0.88)	---	0.82 (0.73, 0.91)	<0.001
≥ 60	0.70 (0.63, 0.77)	---	0.68 (0.60, 0.76)	<0.001
Triglycerides (mg/dL)				
≤ 150	1	---	1	
150–199	1.14 (1.05, 1.25)	---	1.14 (1.05, 1.25)	0.004
≥ 200	1.51 (1.39, 1.63)	---	1.48 (1.35, 1.61)	<0.001
Systolic blood pressure (mm Hg)				
< 120	1	---	1	
120–139	1.07 (0.94, 1.22)	---	1.02 (0.89, 1.16)	0.81
≥ 140	1.33 (1.18, 1.48)	---	1.12 (1.01, 1.26)	0.04
Albumin (g/dL)				
≤ 3.5	1	---	1	
3.6–3.9	0.91 (0.84, 0.98)	---	0.93 (0.86, 1.00)	0.05
≥ 4.0	0.76 (0.69, 0.83)	---	0.76 (0.69, 0.84)	<0.001
Normalized protein catabolic rate (g/kg/d)				
< 0.8	1	---	1	
0.8–1.00	0.82 (0.75, 0.90)	---	0.82 (0.76, 0.90)	<0.001
≥ 1.01	0.84 (0.78, 0.91)	---	0.85 (0.79, 0.92)	<0.001
eGFR ^f^ (mL/min/1.73 m^2^)				
< 7	1	---	1	
7–10	1.25 (1.13, 1.38)	---	1.16 (1.04, 1.29)	0.009
≥ 10	1.10 (0.98, 1.22)	---	1.38 (1.25, 1.53)	<0.001
Hemoglobin (g/dL)				
< 10	1	---	1	
10–11	1.17 (0.98, 1.38)	---	1.17 (0.99, 1.39)	0.18
≥ 11	1.40 (1.21, 1.61)	---	1.11 (0.99, 1.24)	0.09

^a^In 16,297 US adult patients with diabetes mellitus initiating maintenance hemodialysis at a DaVita outpatient facility

^b^Model 1 included demographic and area-level socioeconomic variables

^c^Model 2 was a multivariable model that included all demographic, area-level socioeconomic, and clinical and biometric variables

^d^ *P* values correspond to significance values for Model 2

^e^Compared with white race as the reference category

^f^eGFR, estimated glomerular filtration rate

Poor glycemic control was significantly associated with markers of inflammation. Patients in the second and third tertiles of platelet count had 10 and 15 % higher prevalences of poor diabetes control, respectively, compared with those in the lowest tertile. Patients in the second and third tertiles of white blood cell count had 11 and 18 % higher prevalences of poor control, respectively, compared with those in the lowest tertile. The PR associated with ferritin concentration ≥400 ng/mL was 1.17 (95 % CI, 1.08, 1.27) compared with ferritin concentration <200 ng/mL.

Components of metabolic syndrome were associated with poor glycemic control. Patients with BMI ≥30 kg/m^2^ had a 10 % (95 % CI, 1 %, 21 %) higher prevalence of poor glycemic control compared with those with BMI between 19 and <25 kg/m^2^. Worse lipid metabolism markers were also associated with poor glycemic control. Patients with total cholesterol ≥240 mg/dL and triglycerides ≥200 had adjusted PRs of 1.12 (95 % CI, 1.01, 1.29) and 1.48 (95 % CI, 1.35, 1.61) respectively, whereas patients with HDL ≥60 mg/dL had a 32 % (95 % CI, 24 %, 40 %) lower prevalence of poor glycemic control compared with those with HDL ≤40 mg/dL. Patients whose systolic blood pressure was ≥140 mm Hg had a 12 % (95 % CI, 1 %, 26 %) higher prevalence of poor glycemic control compared with those whose blood pressure was less than 120 mm Hg.

eGFR at initiation of hemodialysis was positively associated with poor glycemic control. Compared with patients with eGFR less <7 mL/min/1.73 m^2^, those with eGFR between 7 and 10 mL/min/1.73 m^2^ had a 16 % (95 % CI, 4 %, 29 %) higher prevalence, and those with eGFR ≥10 mL/min/1.73 m^2^ had a 38 % (95 % CI, 25 %, 53 %) higher prevalence of poor glycemic control. Albumin and nPCR were inversely associated with poor HbA1c control (*P* < 0.05 for both), but hemoglobin was not independently associated with poor glycemic control.

These findings were robust to the different outcome definitions of HbA1c >7.5 % (12 % of patients) and >8 % (8 % of patients) used in sensitivity analyses. The point estimates of PR were similar for all the variables except white blood cell count, which lost statistical significance with a higher cutoff of HbA1c >8 %.

Toward conducting stratified analyses by diabetes type, we found that 627 patients or approximately 4 % of our cohort were presumed to have had type 1 diabetes based on their age. The mean age of patients with type 1 diabetes was 33.0 years (±4.6 years) and that of patients with type 2 diabetes was 66.0 years (±11.4 years). Patients with type 1 diabetes had a 61 % (95 % CI, 4 %, 84 %) higher prevalence of poor HbA1c control compared with those with type 2 diabetes. Findings among patients with type 1 diabetes did not vary from those of the overall cohort with the exception of variables such as age, Native American race, Hispanic ethnicity, and clinical variables such as BMI, white blood cell count, ferritin, HDL, SBP, albumin, and nPCR, all of which were no longer significantly associated with poor HbA1c control (Table 3). Platelet count and eGFR were more strongly associated with poor control among patients with type 1 diabetes compared with those in the overall cohort. We did not find any significant differences in findings among patients with type 2 diabetes compared with those in the overall cohort.

**Table 3:** Prevalence ratios of poor glycemic control (HbA1c >7 % vs. ≤7 %) by diabetes type

Determinants	Type 1^a^	*P*-value	Type 2^b^	*P*-value
Age (years)^c^				
Tertile 1	1		1	
Tertile 2	1.31 (0.95, 1.80)	0.10	0.78 (0.72, 0.84)	<0.001
Tertile 3	1.04 (0.76, 1.70)	0.81	0.57 (0.52, 0.62)	<0.001
Male sex	0.94 (0.71, 1.24)	0.67	1.08 (1.00, 1.16)	0.05
Race/ethnicity^d^				
Native American	1.03 (0.52, 2.05)	0.93	1.47 (1.20, 1.79)	<0.001
Asian	0.48 (0.11, 2.05)	0.32	1.00 (0.80, 1.25)	0.99
Black	0.82 (0.58, 1.16)	0.27	1.04 (0.95, 1.14)	0.44
Pacific Islander	0.78 (0.18, 3.42)	0.75	0.95 (0.61, 1.48)	0.82
Other/Multiracial	0.41 (0.05, 3.07)	0.38	0.79 (0.38, 1.67)	0.53
Hispanic ethnicity	0.75 (0.51, 1.11)	0.15	1.18 (1.06, 1.31)	0.002
Median rent ($)				
≤ 735	1		1	
736–959	0.79 (0.57, 1.08)	0.14	0.94 (0.86, 1.03)	0.18
≥ 960	0.81 (0.53, 1.23)	0.33	0.99 (0.89, 1.10)	0.87
Median household income ($)				
< 38,629	1		1	
38,630–52,301	1.17 (0.79, 1.75)	0.43	1.07 (0.97, 1.19)	0.19
> 52,303	1.02 (0.60, 1.81)	0.94	1.11 (0.97, 1.27)	0.14
% below poverty				
< 12	1		1	
12–21	0.86 (0.56, 1.32)	0.49	1.07 (0.96, 1.20)	0.22
≥ 21	0.86 (0.49, 1.53)	0.61	1.10 (0.94, 1.29)	0.22
% unemployed				
< 5	1		1	
5–7	0.97 (0.68, 1.38)	0.86	1.04 (0.95, 1.13)	0.42
> 7	1.16 (0.79, 1.69)	0.45	1.05 (0.95, 1.16)	0.30
% with < high school education				
< 11.5	1		1	
11.5–19.5	0.83 (0.57, 1.21)	0.33	0.96 (0.87, 1.07)	0.45
≥ 19.5	0.94 (0.67, 1.46)	0.80	0.98 (0.87, 1.11)	0.80
Smoking (current)	1.06 (0.69, 1.61)	0.80	0.97 (0.83, 1.12)	0.67
BMI (kg/m^2^)				
19- < 25	1		1	
25–29	1.23 (0.88, 1.72)	0.28	1.03 (0.93, 1.14)	0.60
≥ 30	0.86 (0.62, 1.21)	0.39	1.14 (1.04, 1.26)	0.008
Platelet count (×10^3^/μL)				
≤ 207	1		1	
208–277	1.68 (1.03. 2.74)	0.04	1.08 (0.99, 1.18)	0.08
≥ 278	2.28 (1.42, 3.63)	<0.001	1.10 (1.01, 1.21)	0.04
White blood cell count (1000 per μL)				
< 6.3	1		1	
6.3–8.2	1.01 (0.71, 1.45)	0.94	1.11 (1.02, 1.22)	0.02
> 8.2	0.97 (0.70, 1.38)	0.86	1.19 (1.08, 1.31)	<0.001
Ferritin (ng/mL)				
< 200	1		1	
200–399	0.90 (0.62, 1.31)	0.59	1.01 (0.93, 1.11)	0.77
≥ 400	1.11 (0.80, 1.55)	0.53	1.15 (1.06, 1.26)	0.001
Total cholesterol (mg/dL)				
≤ 200	1		1	
201–239	1.07 (0.74, 1.54)	0.73	1.12 (0.99, 1.26)	0.06
≥ 240	1.12 (1.09, 1.61)	0.02	1.17 (1.03, 1.34)	0.01
HDL (mg/dL)				
≤ 40	1		1	
40–59	0.93 (0.64, 1.34)	0.70	0.82 (0.72, 0.92)	<0.001
≥ 60	0.72 (0.47, 1.11)	0.14	0.68 (0.60, 0.77)	<0.001
Triglycerides (mg/dL)				
≤ 150	1		1	
150–199	1.23 (0.87, 1.75)	0.25	1.14 (1.04, 1.25)	0.007
≥ 200	1.45 (1.03, 2.05)	0.03	1.48 (1.35, 1.62)	<0.001
Systolic blood pressure (mm Hg)				
< 120	1		1	
120–139	1.34 (0.74, 2.42)	0.33	1.00 (0.88, 1.15)	0.97
≥ 140	1.21 (0.72, 2.05)	0.47	1.12 (0.99, 1.26)	0.07
Albumin (g/dL)				
≤ 3.5	1		1	
3.6–3.9	0.76 (0.58, 1.03)	0.08	0.94 (0.87, 1.02)	0.15
≥ 4.0	0.81 (0.54, 1.22)	0.30	0.76 (0.69, 0.85)	<0.001
Normalized protein catabolic rate (g/kg/d)				
< 0.8	1		1	
0.8–1.00	1.18 (0.84, 1.65)	0.33	0.80 (0.73, 0.88)	<0.001
≥ 1.01	1.09 (0.81, 1.49)	0.57	0.83 (0.77, 0.90)	<0.001
eGFR ^e^ (mL/min/1.73 m^2^)				
< 7	1		1	
7–10	1.59 (1.04, 2.43)	0.03	1.14 (1.01, 1.27)	0.03
≥ 10	1.66 (1.13, 2.43)	0.009	1.36 (1.23, 1.51)	<0.001
Hemoglobin (g/dL)				
< 10	1		1	
10–11	0.95 (0.57, 1.61)	0.86	1.21 (0.98, 1.45)	0.07
≥ 11	1.03 (0.68, 1.57)	0.90	1.45 (1.00, 1.70)	0.05

^a^In 627 US adult patients with type 1 diabetes mellitus initiating maintenance hemodialysis at a DaVita outpatient facility. Multivariable model included all demographic, area-level socioeconomic, and clinical and biometric variables

^b^In 15,670 US adult patients with type 2 diabetes mellitus initiating maintenance hemodialysis at a DaVita outpatient facility. Multivariable model included all demographic, area-level socioeconomic, and clinical and biometric variables

^c^Age breakdown in tertiles was as follows: 18–31 (tertile 1), 32–36 (tertile 2), 37–39 (tertile 3) for patients with type 1 diabetes, and 40–61 (tertile 1), 62–71 (tertile 2), and >71 (tertile 3) for patients with type 2 diabetes

^d^Compared with white race as the reference category

^e^eGFR, estimated glomerular filtration rate

## Discussion

In this large cohort of incident US hemodialysis patients with diabetes mellitus, we found that poor glycemic control, defined as HbA1c >7 %, was more common in younger patients and among Native American and Hispanic patients. Poor glycemic control was further associated with components of metabolic syndrome including higher BMI, unfavorable lipid profile, uncontrolled blood pressure, and with markers of inflammation, malnutrition, and a more serious systemic disease.

The inverse relationship that we found between age and poor glycemic control is consistent with the findings of studies not restricted to the ESRD setting [16, 17]. In stratified analyses by diabetes type, we found that occurrence of diabetes in patients <40 years of age, a proxy for type 1 diabetes, was associated with poor HbA1c control compared with that in patients ≥40 years of age, which was classified as type 2 diabetes. These findings suggest that there may be pathophysiologic differences in relation to glycemic control between type 1 and type 2 diabetes, which are reflected in the differences we observed in the association between age and glycemic control. Behavioral differences may also explain the association between younger age and poorer glycemic control. Older patients may feel more determined and willing to adhere to their medication therapy or to adopt a healthy lifestyle consisting of regular exercise and healthy diet [18]. Another explanation could be that with increasing age, relatively more compliant and appropriate patients are selected to start dialysis treatment, whereas those who are considered less suitable and less likely to maintain diabetic control opt for conservative ESRD treatment without undergoing dialysis [19, 20]. It is also possible that the lower HbA1c levels observed in older patients is due to lower caloric intake and/or malnutrition, which have been shown to be associated with low HbA1c in the elderly, indicative of poor health and frailty [21]. In older patients who were classified as having type 2 diabetes, we found that BMI was positively associated with poor glycemic control while markers for malnutrition such as albumin and nPCR were inversely associated with poor control. However, these variables were not associated with poor glycemic control among younger patients who were classified as having type 1 diabetes. Although variables such as Native American race, Hispanic ethnicity, and clinical variables including white blood cell count, ferritin, HDL, and SBP were not associated with poor glycemic control in patients with type 1 diabetes as they were in the overall cohort, this could be due to small sample size of patients with type 1 diabetes in our cohort (4 % of the overall cohort) and the lack of power to detect true associations.

Given racial and ethnic differences in the development and progression of diabetes complications [22], we expected the prevalence of poor glycemic control to be higher in minorities compared with non-Hispanic whites. However, multivariable-adjusted models demonstrated that the prevalence of poor glycemic control was only higher in Native American and Hispanic patients compared with that in non-Hispanic white patients, after adjusting for five area-level socioeconomic indicators. Interestingly, we found that poor glycemic control was not associated with any of the five area-level socioeconomic variables that were examined in fully adjusted models. Findings were not sensitive to adjustment for several variables that could be hypothesized to lie on the potential causal pathway between socioeconomic factors and poor HbA1c control including BMI, metabolic and inflammatory markers. Our findings contrast with other studies in which associations between socioeconomic factors and poor HbA1c control were found [23, 24]. However, the effects of socioeconomic status may be blunted in an insured population such as that of Medicare beneficiaries with ESRD, who are seen regularly or at least monthly by a nephrologist, in which financial barriers to health care are not as prominent.

Our observation that an atherogenic lipoprotein profile and components of metabolic syndrome, including BMI, HDL, triglycerides, and blood pressure, were associated with poor glycemic control is consistent with earlier studies [11, 25–28]. Since ours is a cross-sectional study, we cannot infer causality; however, either direction of causality would have critical clinical implications in hemodialysis patients with diabetes among whom cardiovascular disease is a major cause of morbidity and mortality [29].

Our data suggest that poor glycemic control is associated with markers of increased inflammation and infection, independent of BMI. These findings are supported by previous studies that have found relations between hyperglycemia and inflammation and endothelial dysfunction [30]. Although it would have been ideal to assess the relation of systemic inflammation with glycemic control using C-reactive protein (CRP) as a marker for inflammation, we were not able to do so due to data on CRP being unavailable for a majority of the patients since it is not measured routinely in the care of these patients.

We found that higher eGFR at initiation of dialysis was associated with poor glycemic control, suggesting that sicker patients in uremic state who initiate dialysis earlier (i.e., with more preserved kidney function) may present with higher HbA1c levels. Previous studies have reported that uremic toxins influence glucose homeostasis by increasing insulin resistance, promoting hepatic gluconeogenesis, and suppressing peripheral glucose utilization in ESRD patients [31]. Another explanation could be that patients with a higher eGFR have lower creatinine production, a marker for lower muscle mass and malnutrition which are known to be involved in the pathogenesis of diabetes [32]. Grootendorst et al*.* [33] found that higher eGFR was associated with a higher mortality and reasoned that while plasma creatinine is determined by GFR and muscle mass, in patients with impaired renal function, such as those with ESRD, muscle mass becomes the more important determinant of plasma creatinine with declining GFR. The authors showed that eGFR was inversely associated with muscle mass and this association was particularly stronger in patients with diabetes [33].

We were not surprised by the finding that higher albumin levels were associated with improved glycemic control. There have been previous reports of excess mortality in ESRD patients being attributed to low serum albumin levels, potentially a proxy for malnutrition, which are independent predictors of morbidity and mortality in this patient population [34, 35]. These findings about protein malnutrition and glycemic control were further substantiated by nPCR data from which we found that patients in the highest tertile of nPCR had better glycemic control compared with those in the lowest tertile, particularly for patients with type 2 diabetes. While low serum album levels may represent malnutrition arising from uremic syndrome, they may also be a marker of comorbidities and inflammation more generally, indicative of a more serious systemic disease [36]. Hence, the association we observed between serum albumin and HbA1c is in line with our findings that support the relation of inflammation with poor glycemic control, and the association between HbA1c and eGFR at initiation of dialysis which may be representative of a more serious disease status.

Whether HbA1c accurately reflects mean blood glucose levels in patients with diabetes on hemodialysis is somewhat controversial, as some would argue that it may not be a reliable marker for long-term glycemic control [37]. Dialysis patients have shorter erythrocyte lifespan, and low concentrations of erythrocytes in those with anemia or the predominance of younger erythrocytes observed in patients who are on iron replacement therapy or erythropoiesis-stimulating agents can result in falsely low HbA1c values, underestimating the patient’s glycemic state [38]. While some studies have advocated the use of glycated albumin and fructosamine as alternative measures of glycemic control in dialysis patients, these markers are easily influenced by various physiological conditions [38]. Moreover, the within-subject variation of fructosamine is higher than that of HbA1c, and the use of fructosamine as a marker for glycemic control would depend on normal serum albumin levels, which are rarely observed in dialysis patients [38]. In the absence of consistent and ample clinical data supporting the use of glycated albumin and fructosamine as potential markers of glycemic control, it would be reasonable to use HbA1c as the reference standard for hemodialysis patients with diabetes.

Our study has some limitations. Due to the cross-sectional design of the study, we cannot establish directionality of the observed associations. Prospective epidemiological studies are needed to address the question of whether these sociodemographic and clinical factors lead to higher HbA1c levels or whether poor glycemic control leads to these risk factors. We also cannot rule out potential residual confounding due to the observational nature of the study. Furthermore, we could not determine and adjust for the severity of comorbidities because these data were abstracted from an administrative database. Lastly, the USRDS does not reliably distinguish between type 1 and type 2 diabetes. We used the occurrence of diabetes in patients of ages <40 and ≥40 years as a surrogate for type 1 and type 2 diabetes respectively, but there is some diagnostic uncertainty in using this imperfect approach to making the distinction between type 1 and type 2 diabetes, especially in light of the fact that there is a growing frequency of type 2 diabetes in younger patients [39] and that patients with type 1 diabetes may have reached ESRD after 40 years of age. Despite these limitations, the study herein takes advantage of two unusually large and detailed data sources to characterize patients according to a wide array of demographic and socioeconomic factors as well as clinical parameters, and to assess associations between these variables and HbA1c control.

## Conclusion

Our findings indicate that while patient’s age, race, ethnicity are risk factors for higher prevalence of poorly controlled HbA1c, most determinants of poor glycemic control are clinically-relevant factors, including BMI, atherogenic lipid profile, high blood pressure, inflammation, and markers indicative of malnutrition and a more serious systemic disease.

## Abbreviations

ACS: American Community Survey
BMI: Body mass index
CMS: Centers for Medicare and Medicaid Services
CRP: C-reactive protein
CI: Confidence interval
eGFR: Estimated glomerular filtration rate
EHR: Electronic health record
ESRD: End-stage renal disease
HbA1c: Glycated hemoglobin
NIDDK: National Institutes of Diabetes and Digestive and Kidney Disease
nPCR: Normalized protein catabolic rate
PR: Prevalence ratio
USRDS: United States Renal Data System
